# Change in Optic Nerve Sheath Diameter and Cerebral Ventricular Shunt Failure in Children

**DOI:** 10.1001/jamanetworkopen.2025.11009

**Published:** 2025-05-16

**Authors:** Adrienne L. Davis, Mark Tessaro, Suzanne Schuh, Armaan K. Malhotra, Maya Sumaida, Magali Gauthey, Onaiza Zahid, Sara Breitbart, Helen M. Branson, Suzanne Laughlin, Brian W. Hanak, Abhaya V. Kulkarni

**Affiliations:** 1 Division of Pediatric Emergency Medicine, Hospital for Sick Children, Toronto, Ontario, Canada; 2 Research Institute, Hospital for Sick Children, University of Toronto, Ontario, Canada; 3 Institute for Health Policy, Management and Evaluation, University of Toronto, Toronto, Ontario, Canada; 4 Department of Paediatrics, Alberta Children’s Hospital, Calgary, Alberta, Canada; 5 Hopital de la Tour, Geneva, Switzerland; 6 University Hospitals Sussex NHS Foundation Trust, West Sussex, England; 7 Division of Neurosurgery, Hospital for Sick Children, Toronto, Ontario, Canada; 8 Division of Neuroradiology, Hospital for Sick Children, Toronto, Ontario, Canada; 9 Department of Pediatric Neurosurgery, Children’s Hospital of Orange County, Orange, California

## Abstract

**Importance:**

Ocular point-of-care ultrasonography (POCUS) may be a clinically useful method to evaluate shunt dysfunction for children with hydrocephalus presenting to the emergency department (ED).

**Objective:**

To evaluate whether a change in the optic nerve sheath diameter (ONSD) from prior asymptomatic baseline was associated with shunt failure.

**Design, Setting, and Participants:**

Participants in this prospective single-center observational cohort study at a tertiary care children’s hospital were enrolled from January 5, 2018, to March 2, 2022. Children 18 years or younger with cerebrospinal fluid (CSF) shunts were consecutively recruited during routine asymptomatic visits in the outpatient neurosurgery clinic. The institution sees approximately 1000 children with shunts. Children with comorbid eye pathologic conditions known to increase the ONSD were excluded, as were those with shunt interventions between baseline and symptomatic ocular POCUS. Data analysis was completed in May 2024.

**Exposures:**

Baseline ocular POCUS was performed on asymptomatic children with CSF shunts recruited in the outpatient neurosurgery clinic; a second ocular POCUS was performed if the patient subsequently presented to the ED with symptoms of shunt failure. Change in ONSD from prior asymptomatic baseline to symptomatic presentation was the main study exposure.

**Main Outcomes and Measures:**

Shunt failure was defined as intraoperative confirmation of inadequate CSF flow through the shunt system associated with identifiable shunt complications, including catheter or valve obstruction, shunt tubing fracture or disconnection, or proximal catheter migration out of the ventricle within 96 hours from presentation to the ED.

**Results:**

Seventy-six pairs of baseline and symptomatic ultrasonograms from 58 patients (mean [SD] age, 6.6 [4.7] years; 36 of 58 boys [62%]) were included. Twenty patients (35%) had 2 or more prior shunt revisions, and 29 (50%) had communicating hydrocephalus. The primary outcome of intraoperatively confirmed shunt failure was observed in 14 of 76 ED patient presentations (18%). The mean (SD) change in ONSD among patients with shunt failure was 0.89 (0.66) mm vs 0.16 (0.40) mm among patients without shunt failure (mean difference, 0.73 mm [95% CI, 0.47-0.99 mm]; *P* < .001). The odds of full shunt failure were 1.4 times higher (95% CI, 1.21-1.78; *P* < .001) for every 0.1-mm increase in ONSD. The area under the receiver operating characteristic curve was 0.86, with an optimal cutoff of 0.4 mm or more, yielding a sensitivity of 0.93, specificity of 0.73, positive predictive value of 0.43, and negative predictive value of 0.98, for a disease prevalence of 15%, with a positive likelihood ratio of 3.39 and negative likelihood ratio of 0.10.

**Conclusions and Relevance:**

In this single-center, prospective cohort study of symptomatic children with CSF shunts, a change in ONSD measured by ocular POCUS was associated with shunt failure. A change in ONSD from prior asymptomatic baseline to symptomatic presentation of less than 0.4 mm identified a population at low risk of true shunt failure. Further research is warranted to validate these findings, the presented change in ONSD threshold, and to risk stratify low-risk patients for cross-sectional neuroimaging.

## Key Points

**Question:**

Is a change in optic nerve sheath diameter measured on ocular point-of-care ultrasonography associated with shunt failure among symptomatic children with cerebrospinal fluid shunts?

**Findings:**

This cohort study found that a change of 0.4 mm or more in optic nerve sheath diameter from prior asymptomatic baseline was associated with shunt failure in symptomatic children. A change below this cutoff had a 98% negative predictive value for shunt failure.

**Meaning:**

This study suggests that a change in the optic nerve sheath diameter from a patient’s own asymptomatic baseline measured by ocular point-of-care ultrasonography may be a helpful noninvasive screening tool for shunt failure among symptomatic children presenting to the emergency department.

## Teaser

This cohort study evaluates whether a change in optic nerve sheath diameter from prior asymptomatic baseline was associated with shunt failure among symptomatic children with cerebrospinal fluid shunts.

## Introduction

The placement of cerebrospinal fluid (CSF) shunts has markedly reduced mortality among children with hydrocephalus since their introduction in the 1950s. However, due to frequent complications, CSF shunts have been described as “among the most failure-prone life-sustaining medical devices implanted in modern medical practice.”^1^ Approximately 30% of shunts placed in children malfunction within the first year after placement, and 5% per year thereafter.^2,3^ Patients commonly present to the emergency department (ED) with nonspecific symptoms, making it challenging to distinguish between a viral illness and life-threatening shunt failure.^4^ Consequently, affected patients undergo multiple computed tomographic (CT) scans and magnetic resonance imagings (MRIs) per year.^5,6^ In addition to the related risks of ionizing radiation exposure and sedation, up to 30% of patients with blocked shunts may not exhibit ventricular dilatation or other signs of increased intracranial pressure (ICP) on neuroimaging.^7,8,9^

The CSF-filled optic nerve sheath is in anatomic continuity with the intracranial compartment and swells with increased ICP, causing elevation of the optic disc. Both optic nerve sheath diameter (ONSD) and optic disc elevation (ODE) can be easily measured with ocular point-of-care-ultrasonography (POCUS).^10,11,12,13^ Compared with CT scan and MRI, ocular POCUS is portable, radiation-free, easily repeatable, and inexpensive. Prior work by our group has shown excellent interrater reliability of ocular POCUS image acquisition and ONSD measurement by ED and neurosurgery clinicians among children with CSF shunts.^14^ If shown to have favorable test characteristics for shunt failure, ONSD measurement via ocular POCUS could minimize further testing in a low-risk population, and/or indicate the urgency of workup for those at risk.

Age-based normal ranges for ONSD exist in healthy children,^15,16^ and ONSDs exceeding upper limits are associated with increased ICP among children without CSF shunts.^12,17^ However, these upper limits have failed to consistently discriminate between patients with and without shunt failure.^18,19^ In a small, retrospective study of patients with CSF shunts as their own controls, 82.8% of patients had an increase in ONSD that correlated with operating room (OR)–confirmed shunt obstruction.^20^ Comparing ocular POCUS ONSD with previous ocular POCUS ONSD using a within-patient design has strong face validity as it emulates decision heuristics used by neuroradiologists and neurosurgeons when assessing neuroimaging results for shunt failure. Cross-sectional neuroimaging, although contributory, has both false positives and false negatives.^21^ Therefore, most diagnostic test studies define true failure based on intraoperative confirmation of inadequate flow of CSF through the shunt system.^9^

Our primary objective was to evaluate whether a change in ONSD from prior asymptomatic baseline among shunted children younger than 19 years presenting to the ED with shunt malfunction symptoms was associated with shunt failure as determined in the OR by the treating neurosurgeon. We hypothesized that a change in the ONSD would be higher among children with shunt malfunction compared with those without.

Secondary objectives were to evaluate (1) the association between the change in sonographic ODE and shunt failure; (2) the test characteristics (sensitivity, specificity, positive predictive value [PPV], negative predictive value [NPV], area under the receiver operating characteristic curve [AUROC]) of a change in ONSD and a change in ODE with respect to shunt failure; and (3) which ONSD parameter was more associated with shunt failure: averaging between the right and left eyes (change in ONSD-M) or using the highest value of the right and left eyes (change in ONSD-H).

## Methods

### Design and Setting

This was a single-center, prospective observational cohort study at a tertiary care children’s hospital from January 5, 2018, to March 2, 2022. This study was approved by the research ethics board of the Hospital for Sick Children and followed the Strengthening the Reporting of Observational Studies in Epidemiology (STROBE) guideline for reporting observational research. Caregivers provided written consent for participants without capacity to consent themselves, and participants with capacity to consent provided their own written consent.

### Population

Participants were 18 years of age or younger with ventriculoperitoneal, ventriculoatrial, ventriculopleural, or cystoperitoneal shunts. Patients were recruited exclusively during asymptomatic visits in the outpatient neurosurgery clinic. We excluded children with comorbid eye pathologic conditions known to increase ONSD (optic neuritis, optic nerve trauma, optic nerve tumor) and those with symptoms of possible shunt failure, including behavior change, bulging fontanelle, swelling along shunt tract, altered level of consciousness, irritability, vomiting, abnormal shunt pump test, accelerated head growth, or headache. Enrolled participants were later removed from analysis if ultrasonographic images were of poor quality such that both ONSD and ODE could not be measured or if a shunt intervention was subsequently found to have occurred between baseline and symptomatic scans.

### Procedure

Baseline ocular POCUS was performed in the neurosurgery clinic when participants were asymptomatic. A second POCUS was performed only if participants subsequently presented to the ED with symptoms of possible shunt failure. Participants were then followed up prospectively to assess whether they were discharged without intervention or taken to the OR and determined to have shunt failure. Patient demographic characteristics, history, symptoms, whether a CT scan or MRI was completed, and ED clinical impression of likelihood of shunt failure (100-point visual analog scale, where higher scores indicated higher clinical suspicion of shunt failure) were collected by research assistants during the ED visit. All ED, neuroradiology and neurosurgical clinical team members were unaware of POCUS ONSD measurements.

### POCUS Image Acquisition and Measurement

All ED and neurosurgery clinicians performing ocular POCUS for the study underwent standardized training.^14^ Clinicians performing ocular POCUS in the ED were masked to the CT scan and MRI results as well as clinical symptoms and examination findings of participants.

All patients were examined while supine with the head of bed at 30°, using an occlusive plastic dressing over a closed eyelid. Images were acquired in the transverse plane with a 5- to 14-MHz high-frequency linear probe.^22,23^ Multiple 6-second video recordings of each eye were captured such that 3 measurements per eye of ONSD and ODE could be averaged from the best still images from different clips. Trained ocular POCUS operators were instructed to capture the optic nerve running parallel to the ultrasonographic beam by asking compliant patients to position their gaze neutrally. For noncompliant patients, the examination was assisted by having a caregiver hold an age-appropriate video to encourage a straight-ahead gaze from the patient. Patient tolerance of the ocular POCUS was measured using a 7-point Likert scale (score range, 1-7, where higher scores indicate higher tolerance)^14^ (eTable 1 in Supplement 1).

The ONSD measurement occurred at a standard depth of 3 mm below the base of the retina, by drawing a perpendicular line at 90° relative to the optic nerve axis. Specifically, the length of the line connecting the transition point between the hypoechoic outer stripe and the hyperechoic retrobulbar fat was measured^24^ (eFigure 1 in Supplement 1). Only images with clear anatomic differentiation of ONSD components were selected for measurement.^14^ Optic disc elevation was measured by drawing a perpendicular line from the base of the retina to the maximal point of disc elevation with the optic nerve at the center of the image. All ocular POCUS images were quality assessed and measured outside of the clinical setting by a single, POCUS fellowship-trained pediatric ED attending physician who was masked to participant clinical presentation, CT scan and MRI findings, and outcome. By ensuring the clinical team had no access to ONSD baseline or symptomatic measurements, we guaranteed that ocular POCUS findings had no influence on the neurosurgeon’s decision to go to the OR. All images were measured using Horos DICOM software, version 3.0 (Horos Project).

### Cross-Sectional Neuroimaging Review

Two pediatric neuroradiologists rereviewed all CT scans and MRIs while masked to the initial report, each other’s interpretation, ocular POCUS findings, and clinical outcomes, to assess whether the studies showed evidence of increased ICP and/or other signs of shunt failure. A positive study result was defined as (1) an increase in the size of the ventricular system (not attributable to another cause) compared with a prior study, or interstitial edema of periventricular tissues with or without effacement of cortical sulci, and/or (2) a shunt that was disconnected or migrated (defined as a new disconnection compared with a prior study and/or the shunt migrated out of the ventricle). Discrepancies in imaging readings were resolved by consensus.

Our primary outcome, full shunt failure (yes or no), was defined by intraoperative confirmation of inadequate flow of CSF through the shunt system associated with identifiable shunt complications, including but not limited to catheter or valve obstruction, shunt tubing fracture or disconnection, or proximal catheter migration out of the ventricular system, within 96 hours from symptomatic presentation to the ED. Given the natural history of rapid neurologic deterioration after true shunt malfunction, most patients presenting to the ED with suspected shunt failure will go to the OR within 24 hours of presentation, and based on review of our institution’s neurosurgical database, all such patients undergo shunt revision within 96 hours of initial presentation.

Participants without full shunt failure were screened for intermittent failure, which was defined as an initial increase in ventricle caliber on cross-sectional neuroimaging results, followed by symptom resolution prior to surgery and subsequent resolution of enlarged ventricles. Participants who were discharged from the ED were followed up to assess whether representation to the ED or OR with shunt malfunction occurred within 96 hours of their ED ocular POCUS.

### Statistical Analysis

Statistical analysis was completed in May 2024. Baseline variables were summarized as counts with percentages for categorical variables and mean (SD) values for continuous variables. Demographic, clinical, and POCUS variables were then compared across the full shunt failure group and the no shunt failure group using χ^2^ tests for categorical variables and 2-sample *t* tests for continuous variables. Nonnormally distributed continuous variables were summarized using median with IQR and compared by Wilcoxon rank sum test.

Several patients had multiple ED presentations during the study period. For these patients, the same baseline ONSD and ODE were used to calculate the change on subsequent ED visits as long as there were no shunt interventions in between ED presentations, which was confirmed through caregiver interview, medical record review, and consultation with neurosurgery. Our primary analysis was to assess whether there were differences in the change in the ONSD and the change in ODE between patients with or without shunt failure. We conducted random intercept mixed-effects linear regression to determine mean differences while accounting for correlation of repeated measures taken from individual patients (patient identifier was used as the random intercept term). The null hypothesis was that there was no mean difference between patients with true shunt failure and those without. Analyses were conducted using R statistical programming language, version 4.3.1 (R Project for Statistical Computing). All *P* values were from 2-sided tests, and results were deemed statistically significant at *P* < .05.

We used univariate logistic regression to determine the odds of failure associated with an increase in the ONSD and ODE by 0.1 mm and plotted a receiver operating characteristic (ROC) curve across different thresholds. We reported the sensitivity and specificity of the threshold value that minimized the difference between true-positive rate and false-positive rate. To evaluate the utility of a dynamic vs a static threshold, an ROC plot was also created for the symptomatic measurement ONSD, without measuring change from asymptomatic baseline.

As patients with intermittent failure may have had increased ICP at the time of ED arrival, in sensitivity analysis, we reassessed the mean differences for change in ODE and change in ONSD and repeated ROC analysis using a composite outcome of either full shunt failure or intermittent failure.

## Results

### Baseline Characteristics

**Figure 1.**
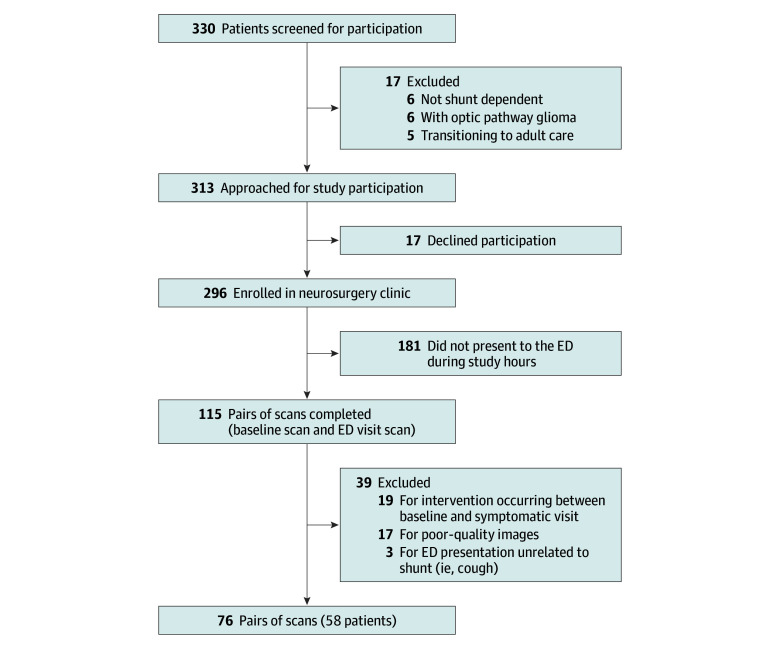
Flow Diagram of Included Participants ED indicates emergency department.

A total of 115 pairs of scans were completed, and 76 pairs of scans from 58 patients (mean [SD] age, 6.6 [4.7] years; 36 of 58 boys [62%] and 22 of 58 [38%] from girls) were included in the analysis (Figure 1; Table 1). Twenty patients (35%) had 2 or more prior shunt revisions, and 29 (50%) had communicating hydrocephalus. Participants with excluded scans had similar mean age, sex, and rate of shunt failure. The median time between asymptomatic baseline ocular POCUS and ED ocular POCUS was 10 months (IQR, 4.8-16.0 months). The mean (SD) baseline (asymptomatic) ONSD for right eyes was 5.5 (0.8) mm and for left eyes was 5.4 (0.8) mm. Among patients without shunt failure, the mean (SD) symptomatic ONSD for right eyes was 5.6 (0.9) mm and for left eyes was 5.6 (0.9) mm. Among patients with shunt failure, the mean (SD) symptomatic ONSD for right eyes was 6.0 (0.5) mm and for left eyes was 5.9 (0.7) mm. A total of 16 of 76 ED patient presentations (21%) resulted in a trip to the OR, and 14 of 76 (18%) had intraoperatively confirmed full shunt failure; an additional 3 patients had intermittent failure.

**Table 1.** Baseline and Clinical Characteristics at ED Presentation Stratified by Presence or Absence of Full Shunt Failure

Characteristic	Patients, No. (%)			*P* value^a^
	Overall (N = 76)	Shunt failure		
		No (n = 62)	Full (n = 14)	
**Patient demographics**				
Age, mean (SD), y	8.1 (4.8)	8.5 (4.6)	6.6 (5.4)	.19
Sex				
Female	26 (34)	20 (32)	6 (43)	.66
Male	50 (66)	42 (68)	8 (57)	
**Clinical characteristics**				
Glasgow Coma Scale in ED^b^				
15	74 (97)	61 (98)	13 (93)	.10
14	1 (1)	1 (2)	0	
13	1 (1)	0	1 (7)	
Symptoms				
Agitation	11 (15)	8 (13)	3 (21)	.69
Vomiting	43 (57)	31 (50)	12 (86)	.03
New neurologic deficit	14 (18)	11 (18)	3 (21)	>.99
Decreased activity	27 (36)	23 (37)	4 (29)	.77
Headache	52 (69)	44 (71)	8 (62)	.73
Positive neuroimaging result (CT scan or MRI)^c^	26 (34)	12 (19)	14 (100)	<.001
Obstructive hydrocephalus	39 (51)	34 (55)	5 (36)	.32
Nonobstructive hydrocephalus	37 (49)	28 (45)	9 (64)	
Hydrocephalus cause^d^				
Congenital	40 (53)	29 (47)	11 (79)	.06
Tumor	16 (21)	14 (23)	2 (14)	.75
Spina bifida or myelomeningocele	10 (13)	9 (15)	1 (7)	.77
Other	20 (26)	19 (31)	1 (7)	.14
Symptom duration, median (IQR), d	1.0 (1.0-4.5)	1.0 (1.0-5.5)	3.5 (1.3-4.0)	.09
ED clinician estimation of shunt malfunction on 100-point visual analog scale, mean (SD)^e^	38.3 (23.3)	35.7 (21.3)	48.3 (28.7)	.17

Abbreviations: CT, computed tomography; ED, emergency department; MRI, magnetic resonance imaging.

^a^ *P* values reflect results of χ^2^ tests for categorical variables and 2-sample *t* tests for continuous variables.

^b^ The range is from 3 to 15, where 3 is the worst and 15 is the best. It is calculated based on eye opening, verbal response, and motor response.

^c^ Positive neuroimaging result defined as: (1) increase in size of ventricular system (not attributable to another cause) compared with prior study, or interstitial edema of periventricular tissues with or without effacement of cortical sulci, and/or (2) a new disconnection of shunt or shunt migration out of the ventricle.

^d^ Hydrocephalus causes are not mutually exclusive; therefore, proportions exceed 100%.

^e^ Higher scores indicate higher suspicion of shunt failure.

There were no statistically significant differences in most demographic and clinical characteristics between patients with and patients without shunt failure (Table 1). Patients with shunt failure were more likely than those without to vomit (86% [12 of 14] vs 50% [31 of 62]; *P* = .03) and to have a CT scan or MRI result with signs of shunt failure (100% [14 of 14] vs 19% [12 of 62]; *P* < .001).

### Association Between Change in POCUS Measures and Shunt Failure

**Figure 2.**
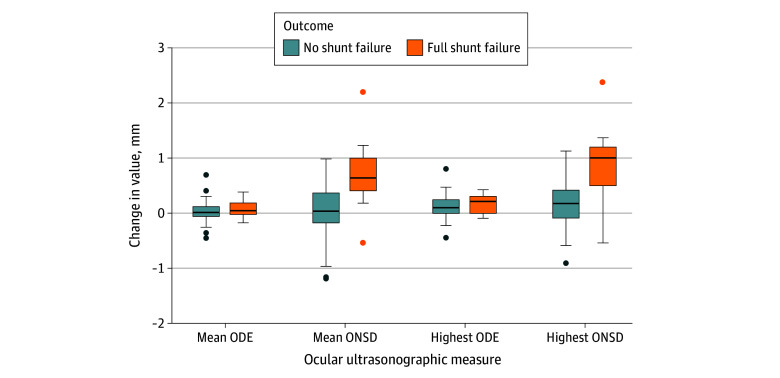
Differences in Change in Optic Nerve Sheath Diameter (ONSD) and Change in Optic Disc Elevation (ODE) Among Patients With or Without Shunt Failure Box plots depicting differences between full shunt failure and no failure and ocular ultrasonographic measures (ODE and ONSD) with corresponding 95% CIs. Mean measures averaged the change in ONSD and change in ODE of the left and right eyes. Highest measures took the highest change in ONSD or change in ODE of the right and left eyes. Vertical lines indicate 95% CIs. The middle line inside the box indicates the median. The top of the box and the bottom of the box indicate the upper and lower quartiles, respectively, and the dots indicate outliers.

We evaluated 2 different measures of the change in the ONSD and the change in ODE: mean measures combined the mean of left and right eyes (change in the ONSD-M and change in ODE-M) and high measures used the highest change in the ONSD or change in ODE of the right and left eyes (change in the ONSD-H or change in ODE-H). Both the mean and the high change in the ONSD yielded larger positive values among patients with shunt failure compared with those without; however, the same was not found for the change in ODE (Figure 2). The mean (SD) change in ONSD-M among patients with shunt failure was 0.73 (0.62) mm, and the mean (SD) change in ONSD-M among those without shunt failure was 0.02 (0.45) mm (mean difference, 0.71 mm [95% CI, 0.42-0.99 mm]; *P* < .001) (Table 2). Similarly, the mean (SD) change in ONSD-H among patients with shunt failure was 0.89 (0.66) mm vs 0.16 (0.40) mm among patients without shunt failure (mean difference, 0.73 mm [95% CI, 0.47-0.99 mm]; *P* < .001). Mean differences were not statistically significant for either change in ODE measure.

**Table 2.** Mean Differences Between Statistical Testing Performed for Ocular Ultrasonographic Measures Across Shunt Failure Categories

Measure	Overall (N = 76)	Shunt failure		Mean difference (95% CI)	*P* value
		No (n = 62)	Full (n = 14)		
Mean difference in ONSD (SD), mm^a^	0.15 (0.56)	0.02 (0.45)	0.73 (0.62)	0.71 (0.42 to 0.99)	<.001
Highest difference in ONSD (SD), mm^b^	0.29 (0.53)	0.16 (0.40)	0.89 (0.66)	0.73 (0.47 to 0.99)	<.001
Mean difference in ODE (SD), mm^a^	0.03 (0.19)	0.02 (0.19)	0.08 (0.17)	0.06 (−0.05 to 0.16)	.28
Highest difference in ODE (SD), mm^b^	0.12 (0.20)	0.11 (0.20)	0.18 (0.18)	0.07 (−0.04 to 0.18)	.21

Abbreviations: ODE, optic disk elevation; ONSD, optic nerve sheath diameter.

^a^ Mean difference is the mean change in ONSD of the right and left eyes.

^b^ The highest difference is the highest change in ONSD between the right and left eyes.

**Figure 3.**
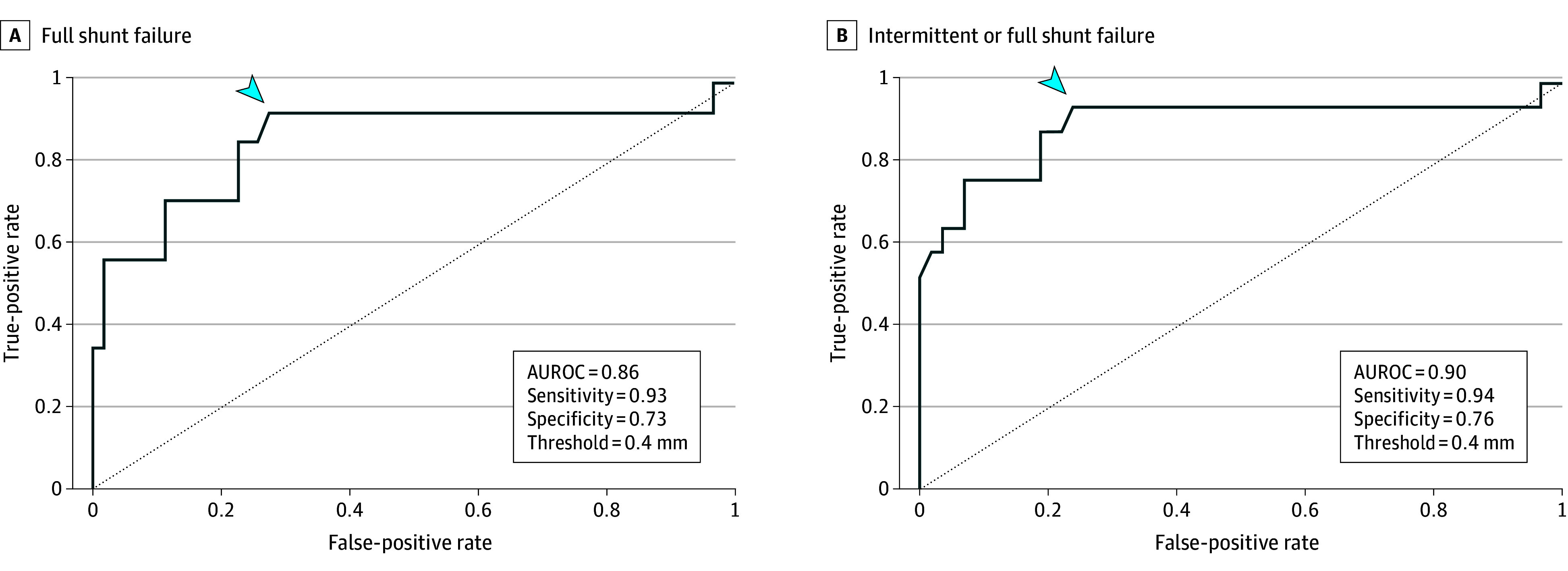
Receiver Operating Characteristic Curve: Change in Optic Nerve Sheath Diameter (ONSD) and Shunt Failure Receiver operating characteristic curve depicting test properties of the highest change measure in ONSD from either eye using the outcome of full shunt failure (A) and intermittent or full shunt failure (B). Additionally, the threshold value maximizing the difference between true-positive rate and false-positive rate (arrowhead) is presented. AUROC indicates area under the receiver operating characteristic curve.

The odds of full shunt failure were 1.4 (95% CI, 1.2-1.8) times higher for every 0.1-mm increase in the change in the ONSD (*P* < .001) and, equivalently, 35 (95% CI, 6-328) times higher for every 1-mm increase in the change in the ONSD (*P* < .001) (eTable 2 in Supplement 1). The AUROC was 0.86, with a cutoff of 0.4 mm or more, which maximized the positive likelihood ratio. This cutoff yielded a sensitivity of 0.93, specificity of 0.73, PPV of 0.43, NPV of 0.98, positive likelihood ratio of 3.39, and negative likelihood ratio of 0.1. eFigure 2 in Supplement 1 demonstrates worse discrimination when evaluating shunt failure using a static ONSD-H threshold at ED assessment (AUROC, 0.68). A sensitivity analysis of patients presenting with either intermittent or full shunt failure revealed the same ideal cutoff of the change in ONSD-H of 0.4 mm or more and yielded a sensitivity of 0.94, specificity of 0.76, PPV of 0.53, and NPV of 0.98, with an AUROC of 0.90 (Figure 3).

### Elaboration of False-Negative Results

eTable 3 in Supplement 1 shows the contingency table results for the change in ONSD-H vs full shunt failure (primary outcome) and full or intermittent shunt failure (sensitivity analysis) using the ROC cut point of 0.4 mm or more. One patient with full shunt failure was missed by using the change in ONSD (a 16-year-old boy with a ventriculoperitoneal shunt for management of obstructive hydrocephalus from a tumor, with 4 prior shunt revisions). He presented with a 3-day history of headache and new nocturnal enuresis. His baseline ultrasonography was done more than 28 months prior to his symptomatic scan with multiple interim ED visits but no shunt intervention. On further review, at the time of his baseline ultrasonography, he had chronic intermittent headache and nausea thought to be not associated with his shunt, with stable neuroimaging results.

## Discussion

We found that patients presenting to the ED with a change in the ONSD less than 0.4 mm from prior asymptomatic baseline had a low likelihood of full shunt failure, with an AUROC of 0.86 and NPV of 98%. If confirmed in future multisite studies, this finding likely represents a low-risk population that may not need immediate neuroimaging and can be safely observed or discharged home with close follow-up. The ability of this test and cutoff to discriminate between children with and children without shunt failure increased further when including patients with both full and intermittent failure (AUROC, 0.90).

Previous literature highlights the imperfect accuracy of traditional imaging in identifying shunt failure. Although noncontrast CT scan is the most common test to assess CSF shunt malfunctions, a recent systematic review of diagnostic modalities for shunt malfunction in children highlighted the limitations of this practice.^9^ The positive likelihood ratio of CT scans varied widely among the studies, ranging from 1.3 to 22.9, making it difficult to assess the true effectiveness of CT scans in confirming CSF shunt malfunctions. For a child with a positive CT scan, the posttest probability of shunt malfunction ranged from 23% to 84%. Pooled MRI sensitivity was 57% and specificity was 93%. Better tools are needed to assist with ruling in and out shunt failure and to identify children for whom traditional imaging may not be necessary.

Prior studies evaluating ONSD measurement on POCUS to predict pediatric shunt failure have yielded mixed results, perhaps in part due to older ultrasonographic technology with limited resolution for distinguishing the optic nerve from its surrounding sheath,^16,17,18,19^ as well as methodological discrepancies in ONSD measurement technique across studies.^24^ Age-based ONSD upper limit cutoffs derived from healthy controls had poor sensitivity, likely due to larger baseline ONSD ranges among children with shunts.^18,19,20^ Similarly, we found that the youngest participants in our study also had ONSD ranges comparable with those of their older counterparts (eFigure 3 in Supplement 1). Given that patients with shunts have varying degrees of shunt dependence, differences in brain compliance, different shunt valve pressure and flow settings, and variability with respect to their tolerance to changes in ICP, it is not surprising that this population would have significant variability in baseline ONSD measurements. Therefore, it makes intuitive sense to control for this variability using a within-patient approach to change in ONSD interpretation rather than a healthy control–derived static threshold. We corroborated this by examining a static threshold for ONSD-H and demonstrated worse discrimination compared with a change in the ONSD for evaluation of shunt failure.

McAuley et al^20^ retrospectively examined the change in the ONSD among 32 children with CSF shunts who underwent serial ocular POCUS examinations when asymptomatic and symptomatic. In their sample, 83% of the children (24 of 29) had a change in ONSD that correlated positively with shunt block in the OR. A total of 66% of patients (19 of 29)s had no change in ventricular configuration on CT scan or MRI findings. Although our study did not include patients with static CT scans or MRI and true shunt failure, this has been previously described.^21,25^ Future larger-scale research is needed to determine whether change in ONSD can identify patients with shunt failure missed by CT scan or MRI.

We found that both the mean change in ONSD-M and the highest change in ONSD-H were associated with shunt failure. In clinical practice, we propose using the more conservative change in ONSD-H because it maximizes sensitivity for capturing this potentially life-threatening condition. Larger-scale research is needed to determine if one truly outperforms the other in estimating shunt failure.

### Limitations

This study has several limitations. Despite being the largest prospective study, to our knowledge, comparing preoperative change in ONSD using a within-patient design, this was a relatively modest sample from a single center. As 100% of participants with shunt failure were identified by cross-sectional imaging, we cannot comment on whether a change in ONSD on POCUS can identify shunt failure missed on CT scan or MRI. In addition, we did not screen participants for the development of ocular pathologic conditions throughout the course of study that may have been associated with ONSD during the symptomatic scan. Future research should screen for confounders at the time of each ocular POCUS scan. Furthermore, a change in ONSD is a known proxy for increased ICP, which is not limited to shunt malfunction. It is possible that participants with true increased ICP had a cause other than shunt malfunction. However, we propose that in using a change in ONSD as a screening test and maximizing sensitivity, we have identified a group of patients who require further investigation, observation, or management vs those who might not. We did not have the power to adjust our analysis for potentially important covariates of shunt failure. We used a single expert measuring ONSD and ODE. Despite excellent interrater reliability in measurement of ONSD,^14^ further research is needed to determine whether bedside measurement of ONSD by multiple ED and/or neurosurgery clinicians yields similar results. Furthermore, some participants were excluded because of missed shunt interventions between the baseline and symptomatic ultrasonograms, often occurring overnight. As patients presenting overnight may be different from their daytime counterparts, future research should allow for 24-hour ocular POCUS access in the ED. Finally, our primary analysis did not account for patients with intermittent shunt failure. As change in ONSD may be best used as a screening tool for patients with suspected shunt failure, this subgroup should be accounted for in future diagnostic test studies.

## Conclusions

In this cohort study of children with CSF shunts, a change in ONSD was associated with shunt failure, while a change in ODE was not. Lack of ODE change among patients with shunts should not provide clinical reassurance. Further multisite research is warranted to validate these findings and the presented change in ONSD threshold, as well as to risk stratify patients at low risk for cross-sectional neuroimaging.

## Notes

Supplement 1. **eTable 1**. Seven-Point Likert Scale Depicting Patient Tolerance of Ocular Point-Of-Care Ultrasound for Left and Right Eyes (n=58 Patients) **eTable 2. **Univariate Logistic Regression Results Depicting Odds Ratio (OR) for Full Shunt Failure Associated With 0.1 mm, 1 mm and Optimal Threshold of ≥0.40 mm Increase in Highest ΔONSD (ΔONSD-H) and Highest ΔODE (ΔODE-H) Measures of the Right OR Left Eye **eTable 3.** Contingency Table Results Using Optimized Threshold Value for Delta Optic Nerve Sheath Diameter (ΔONSD) From Receiver Operating Characteristic Curve to Predict Shunt Failure **eFigure 1.** (A) Probe Orientation; (B) Optic Nerve Sheath Diameter (ONSD) Measurement; (C) Optic Disc Elevation (ODE) Measurement **eFigure 2.** Receiver Operating Characteristic Curve Depicting Test Properties of the Highest Static ONSD Measure From Either Eye at Emergency Department Presentation Using Outcome of Full Shunt Failure **eFigure 3.** (A) Relationship Between Age at Emergency Department Assessment (Years) and the Highest Delta Measurement in ONSD Taken From Either Right or Left Eye; (B) Relationship Between Age at Baseline and Average Baseline ONSD Between Right and Left Eyes

Supplement 2.
Data Sharing Statement
