# Supplementary material for: Change in Optic Nerve Sheath Diameter and Cerebral Ventricular Shunt Failure in Children
Source: JAMA Netw Open. 2025 May 16;8(5):e2511009. doi: 10.1001/jamanetworkopen.2025.11009 (PMC12084842; doi:10.1001/jamanetworkopen.2025.11009)
Supplement: Supplement 1. — eTable 1. Seven-Point Likert Scale Depicting Patient Tolerance of Ocular Point-Of-Care Ultrasound for Left and Right Eyes (n=58 Patients) eTable 2. Univariate Logistic Regression Results Depicting Odds Ratio (OR) for Full Shunt Failure Associated With 0.1 mm, 1 mm and Optimal Threshold of ≥0.40 mm Increase in Highest ΔONSD (ΔONSD-H) and Highest ΔODE (ΔODE-H) Measures of the Right OR Left Eye eTable 3. Contingency Table Results Using Optimized Threshold Value for Delta Optic Nerve Sheath Diameter (ΔONSD) From Receiver Operating Characteristic Curve to Predict Shunt Failure eFigure 1. (A) Probe Orientation; (B) Optic Nerve Sheath Diameter (ONSD) Measurement; (C) Optic Disc Elevation (ODE) Measurement eFigure 2. Receiver Operating Characteristic Curve Depicting Test Properties of the Highest Static ONSD Measure From Either Eye at Emergency Department Presentation Using Outcome of Full Shunt Failure eFigure 3. (A) Relationship Between Age at Emergency Department Assessment (Years) and the Highest Delta Measurement in ONSD Taken From Either Right or Left Eye; (B) Relationship Between Age at Baseline and Average Baseline ONSD Between Right and Left Eyes [file jamanetwopen-e2511009-s001.pdf]

Supplementary Online Content

Davis AL, Tessaro M, Schuh S, et al. Change in optic nerve sheath diameter and cerebral ventricular shunt failure in children. *JAMA Netw Open.* 2025;8(5):e2511009. doi:10.1001/jamanetworkopen.2025.11009

eTable 1. Seven-Point Likert Scale Depicting Patient Tolerance of Ocular Point-Of-Care Ultrasound for Left and Right Eyes (n=58 Patients)

eTable 2. Univariate Logistic Regression Results Depicting Odds Ratio (OR) for Full Shunt Failure Associated With 0.1 mm, 1 mm and Optimal Threshold of ≥0.40 mm Increase in Highest ΔONSD (ΔONSD-H) and Highest ΔODE (ΔODE-H) Measures of the Right OR Left Eye

eTable 3. Contingency Table Results Using Optimized Threshold Value for Delta Optic Nerve Sheath Diameter (ΔONSD) From Receiver Operating Characteristic Curve to Predict Shunt Failure

eFigure 1. (A) Probe Orientation; (B) Optic Nerve Sheath Diameter (ONSD) Measurement; (C) Optic Disc Elevation (ODE) Measurement

eFigure 2. Receiver Operating Characteristic Curve Depicting Test Properties of the Highest Static ONSD Measure From Either Eye at Emergency Department Presentation Using Outcome of Full Shunt Failure

eFigure 3. (A) Relationship Between Age at Emergency Department Assessment (Years) and the Highest Delta Measurement in ONSD Taken From Either Right or Left Eye; (B) Relationship Between Age at Baseline and Average Baseline ONSD Between Right and Left Eyes

This supplementary material has been provided by the authors to give readers additional information about their work.

eTable 1. Seven-Point Likert Scale Depicting Patient Tolerance of Ocular Point-Of-Care Ultrasound for Left and Right Eyes (n=58 Patients)

**Ocular Ultrasound Tolerance**	
**Left Eye**	Counts n (%)
Vigorous movement of body and eyes	3 (7.0)
Moving body and eyes >50% of the time	1 (2.3)
Moving body and eyes <50% of the time	2 (4.7)
Still body, moving eyes >50% of the time	5 (11.6)
Still body, moving eyes <50% of the time	8 (18.6)
Still body and eyes but not following orders about eye motion	9 (20.9)
Still body and eyes, following orders about eye motion	15 (34.9)
**Right Eye**	
Vigorous movement of body and eyes	1 (2.3)
Moving body and eyes >50% of the time	1 (2.3)
Moving body and eyes <50% of the time	2 (4.7)
Still body, moving eyes >50% of the time	6 (14.0)
Still body, moving eyes <50% of the time	8 (18.6)
Still body and eyes but not following orders about eye motion	9 (20.9)
Still body and eyes, following orders about eye motion	16 (37.2)

© 2025 Davis AL et al. *JAMA Network Open.*

eTable 2. Univariate Logistic Regression Results Depicting Odds Ratio (OR) for Full Shunt Failure Associated With 0.1 mm, 1 mm and Optimal Threshold of ≥0.40 mm Increase in Highest ΔONSD (ΔONSD-H) and Highest ΔODE (ΔODE-H) Measures of the Right OR Left Eye. Additionally, sensitivity analysis results are presented for patients with either intermittent or full shunt failure.

**Outcome**	**Full Shunt Failure Primary Outcome)**			**Full or Intermittent Failure (Sensitivity Analysis)**		
**Ocular Ultrasound Measure**	Estimate (OR)	95% CI	p-value	Estimate (OR)	95% CI	p-value
**Δ ONSD-H (per 0.1 mm)**	1.43	1.21 – 1.78	**<0.001**	1.59	1.30 – 2.10	**<0.001**
**Δ ONSD-H (per 1 mm)**	35.43	6.49 – 327.77		103.67	13.93 – 1665.26	
**Δ ONSD of ≥+0.40 mm**	29.42	5.29 – 553.78		43.00	7.81 – 808.05	
**Δ ODE-H (per 0.1 mm)**	1.20	0.89 – 1.65	0.23	1.31	0.99 – 1.81	0.08
**Δ ODE-H (per 1 mm)**	6.21	0.32 – 149.20		14.84	0.87 – 369.59	

© 2025 Davis AL et al. *JAMA Network Open.*

eTable 3. Contingency Table Results Using Optimized Threshold Value for Delta Optic Nerve Sheath Diameter (ΔONSD) From Receiver Operating Characteristic Curve to Predict Shunt Failure. Sensitivity analysis contingency results using full or intermittent obstruction outcome presented below. Optimal threshold for highest ΔONSD (ΔONSD-H) between eyes (≥0.40) derived from ROC analysis.

Labels	Test + (ΔONSD-H ≥0.40)	Test – (ΔONSD-H <0.4)	Overall
**Primary Analysis: Shunt Failure**			
**Failure**	13	1	14
**No Failure**	17	45	62
**Overall**	30	46	76
**Sensitivity Analysis: Full or Intermittent Failure**			
**Failure**	16	1	17
**No Failure**	14	45	59
**Overall**	30	46	76

© 2025 Davis AL et al. *JAMA Network Open.*

eFigure 1. (A) Probe orientation: High frequency linear probe was oriented parallel to the optic nerve in transverse plane. (B) Optic Nerve Sheath Diameter (ONSD) Measurement: ONSD was measured 3mm behind the globe, from where the outer hypoechoic border meets the retrobulbar fat to the other contralateral hypoechoic border. (C) Optic Disc Elevation (ODE) measurement: ODE was measured by drawing a perpendicular line from the base of the retina to the maximal point of disc elevation when the optic nerve was at the center of the image.

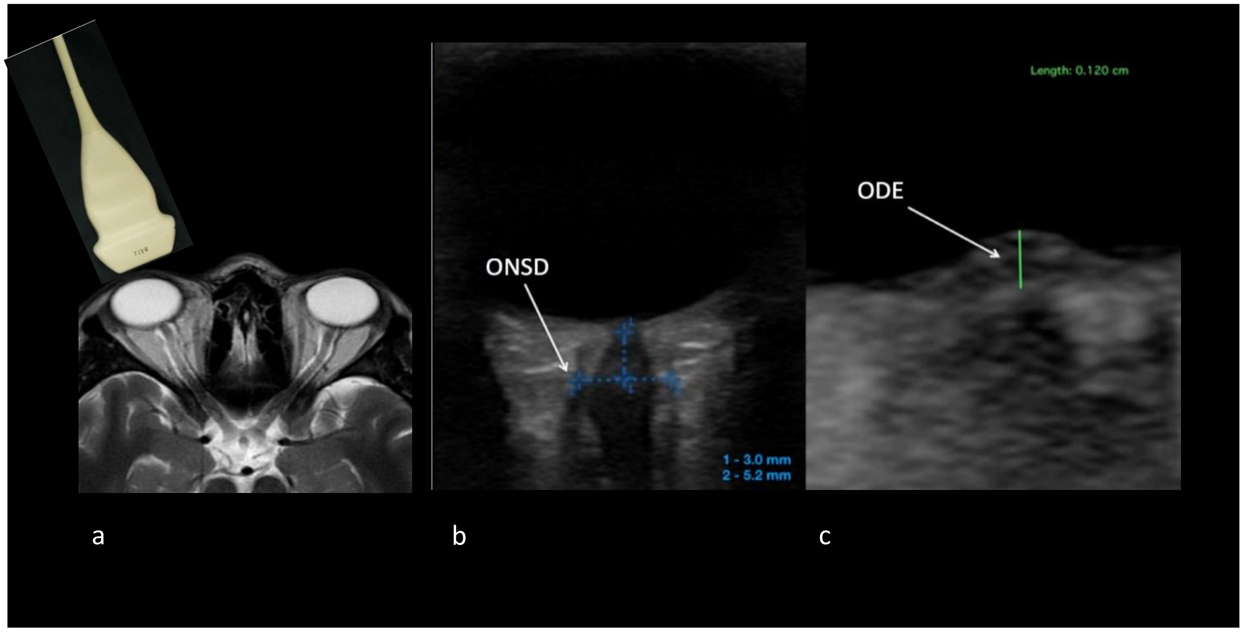

© 2025 Davis AL et al. *JAMA Network Open.*

eFigure 2. Receiver Operating Characteristic Curve Depicting Test Properties of the Highest Static ONSD Measure From Either Eye at Emergency Department Presentation Using Outcome of Full Shunt Failure. We additionally present the threshold value minimizing the difference between true positive rate and false positive rate (blue arrow).

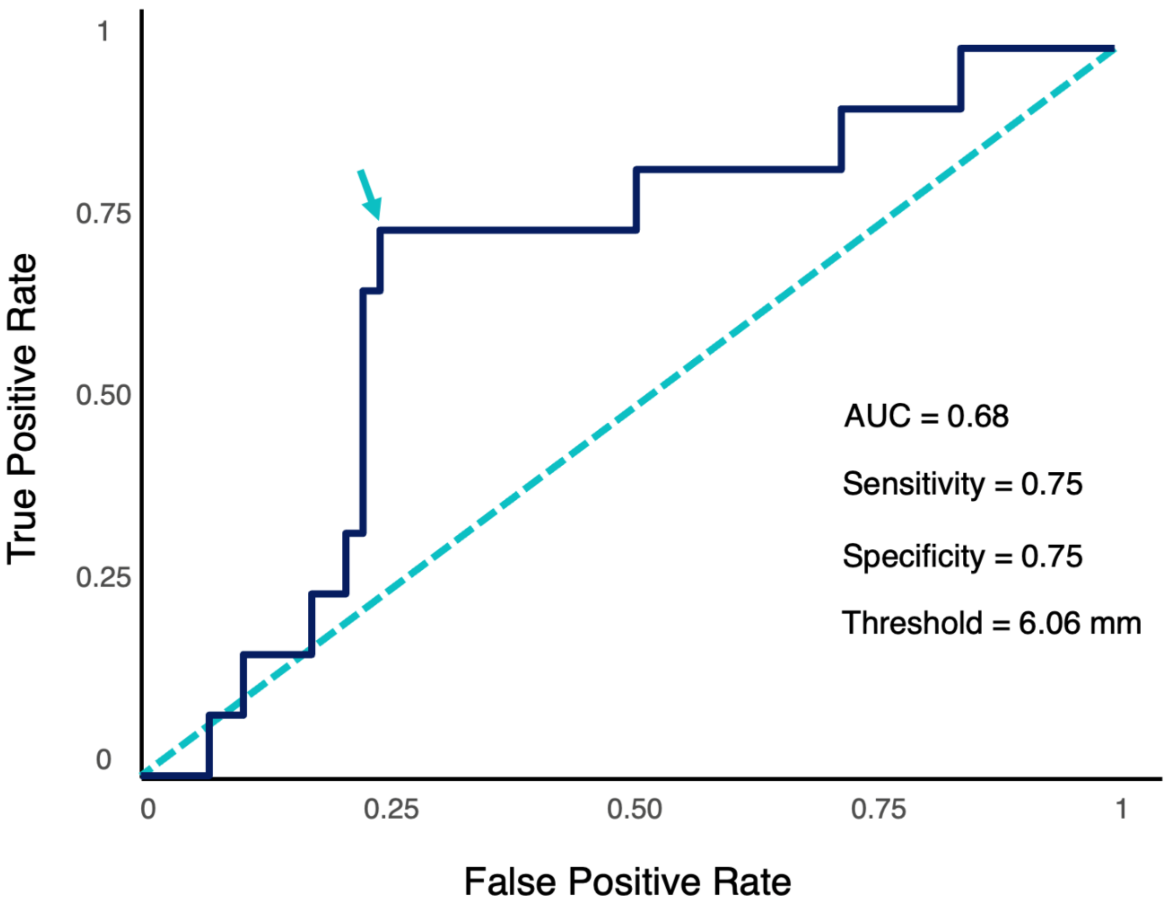

© 2025 Davis AL et al. *JAMA Network Open.*

eFigure 3. (A) Relationship Between Age at Emergency Department Assessment (Years) and the Highest Delta Measurement in ONSD Taken From Either Right or Left Eye. (B) Relationship Between Age at Baseline and Average Baseline ONSD Between Right and Left Eyes.

a)

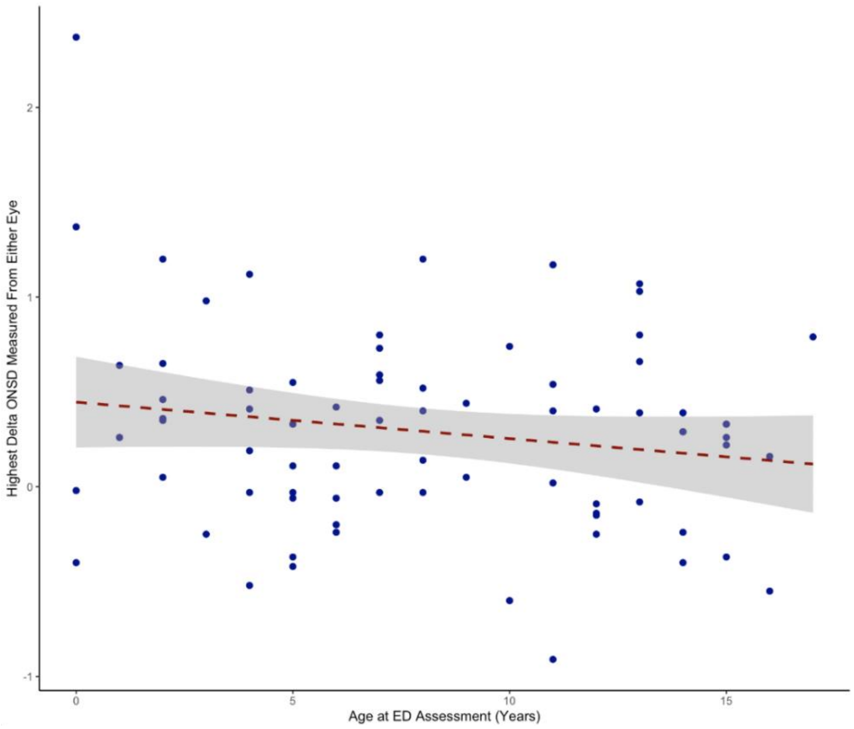

b)

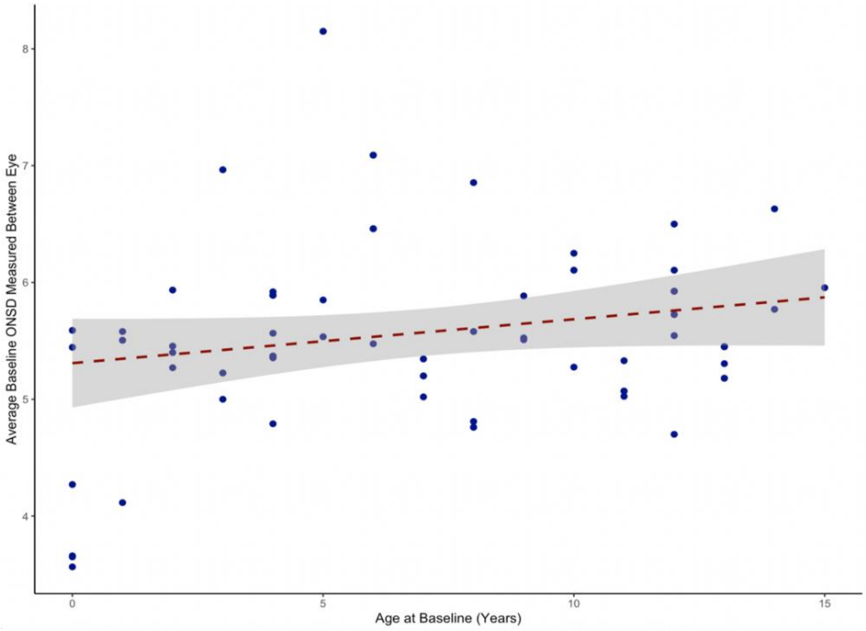

© 2025 Davis AL et al. *JAMA Network Open.*
