# Supplementary material for: Change in Optic Nerve Sheath Diameter and Cerebral Ventricular Shunt Failure in Children
Source: JAMA Netw Open. 2025 May 16;8(5):e2511009. doi: 10.1001/jamanetworkopen.2025.11009 (PMC12084842; doi:10.1001/jamanetworkopen.2025.11009)
Supplement: Supplement 2. — Data Sharing Statement [file jamanetwopen-e2511009-s002.pdf]

Data Sharing Statement

Davis. Change in Optic Nerve Sheath Diameter and Cerebral Ventricular Shunt Failure in Children. *JAMA Netw Open*. Published May 16, 2025.
doi:10.1001/jamanetworkopen.2025.11009

Data
Data available: Yes
Data types: Deidentified participant data, Data dictionary
How to access data: Data will be provided at discretion of PI and provided institutional approval is granted.
When available: With publication

Supporting Documents
Document types: None

Additional Information
Who can access the data: Researchers whose proposed use of the data has been approved.
Types of analyses: Systematic reviews and meta analyses Multisite retrospective studies
Mechanisms of data availability: With investigator support, after approval of a proposal and with a data access agreement
